# Self‐Identified African Americans and prostate cancer risk: West African genetic ancestry is associated with prostate cancer diagnosis and with higher Gleason sum on biopsy

**DOI:** 10.1002/cam4.2434

**Published:** 2019-09-30

**Authors:** William E. Grizzle, Rick A. Kittles, Soroush Rais‐Bahrami, Ebony Shah, George W. Adams, Mark S. DeGuenther, Peter N. Kolettis, Jeffrey W. Nix, James E. Bryant, Ravi Chinsky, James E. Kearns, Kerry Dehimer, Norma Terrin, Hong Chang, Sandra M. Gaston

**Affiliations:** ^1^ Department of Pathology and Surgery University of Alabama at Birmingham Birmingham AL USA; ^2^ Department of Population Sciences City of Hope Duarte CA USA; ^3^ Department of Urology University of Alabama at Birmingham Birmingham AL USA; ^4^ Urology Centers of Alabama Homewood AL USA; ^5^ Tufts Medical Center Boston MA USA; ^6^ Department of Radiation Oncology University of Miami Miller School of Medicine Miami FL USA

**Keywords:** African American, prostate biopsy, prostate cancer, West African ancestry

## Abstract

Concerns about overtreatment of clinically indolent prostate cancer (PrCa) have led to recommendations that men who are diagnosed with low‐risk PrCa be managed by active surveillance (AS) rather than immediate definitive treatment. However the risk of underestimating the aggressiveness of a patient's PrCa can be a significant source of anxiety and a barrier to patient acceptance of AS. The uncertainty is particularly keen for African American (AA) men who are about 1.7 times more likely to be diagnosed with PrCa than European American (EA) men and about 2.4 times more likely to die of this disease. The AA population, as many other populations in the Americas, is genetically heterogeneous with varying degrees of admixture from West Africans (WAs), Europeans, and Native Americans (NAs). Recommendations for PrCa screening and management rarely consider potential differences in risk within the AA population. We compared WA genetic ancestry in AA men undergoing standard prostate biopsy who were diagnosed with no cancer, low‐grade PrCa (Gleason Sum 6), or higher grade PrCa (Gleason Sum 7‐10). We found that WA genetic ancestry was significantly higher in men who were diagnosed with PrCa on biopsy, compared to men who were cancer‐negative, and highest in men who were diagnosed with higher grade PrCa (Gleason Sum 7‐10). Incorporating WA ancestry into the guidelines for making decisions about when to obtain a biopsy and whether to choose AS may allow AA men to personalize their approach to PrCa screening and management.

## Graphical abstract

African American men are at higher risk for prostate cancer but public health recommendations rarely consider potential differences in risk within the African American population. We found that in self‐identified African Americans, West African genetic ancestry was significantly higher in men who were diagnosed with PrCa on biopsy, compared to men who were cancer negative, and highest in men who were diagnosed with higher grade PrCa (Gleason Sum 7‐10). Incorporating West African ancestry into prostate cancer clinical guidelines may allow African American men to personalize their approach to screening and management.

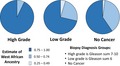

## 1 INTRODUCTION

Well‐publicized concerns about overtreatment of clinically indolent prostate cancer (PrCa) have led to recommendations that men who are diagnosed with low‐risk PrCa be managed by active surveillance (AS) rather than immediate definitive treatment. By deferring PrCa treatment until there is evidence of progression, the patient avoids the potential negative complications of surgery or radiation but accepts the risk that the aggressiveness of his PrCa may have been underestimated. This risk of underestimating a patient's PrCa can be a significant source of anxiety and a barrier to patient acceptance of AS.^1^, ^2^, ^3^ The uncertainty about AS is particularly keen for African American (AA) men. Both incidence and mortality data show that the burden of PrCa is greater for AAs than for European Americans (EAs), with AAs about 1.7 times more likely to be diagnosed with PrCa than EAs and about 2.4 times more likely to die of this disease.^4^, ^5^ Socioeconomic factors contribute to this disparity, but do not fully account for the observation that AA men are more likely than others to be diagnosed with more aggressive and life‐threatening forms of PrCa.^6^, ^7^ Recent studies suggest that AS criteria may need to be modified for AA men^8^, ^9^, ^10^ but there is no consensus.

The AA population, as many other populations in the Americas, is genetically heterogeneous with varying degrees of admixture from West Africans (WAs), Europeans (EUs), and Native Americans (NAs). Recommendations for PrCa screening and management rarely consider potential differences in risk within the AA population. To the extent that genetic factors contribute to the increased risk of PrCa in AA, WA genetic ancestry could potentially be used as a marker to better estimate an AA man's personal PrCa risk. In this study, we tested the hypothesis that in AA men, WA genetic ancestry is associated with higher risk cancer on prostate biopsy.

## 2 METHODS

This study was approved by the University of Alabama at Birmingham IRB and by the Western IRB. Prostate biopsy patients were enrolled as study subjects by urologists affiliated with the University of Alabama at Birmingham and Urology Centers of Alabama. Prior to biopsy, consecutive eligible patients were asked if they wished to participate in the study and if they agreed they were enrolled with written informed consent. Elevated serum prostate‐specific antigen (PSA) was the primary reason for the referral to a urologist for a diagnostic biopsy. A standard‐of‐care ultrasound‐guided 12 core systematic biopsy was used to establish PrCa status as determined by a genitourinary pathologist examination of the hematoxylin and eosin (H&E)‐stained biopsy slides. Demographic and clinical data were obtained by medical record review.

DNA prepared from biopsy cores with no histological evidence of cancer was used for ancestry genotyping. Variations in the distribution of single‐nucleotide polymorphisms (SNPs) have been shown to differentiate human populations^11^ and panels of ancestry informative markers (AIMs) have been developed to distinguish populations of different biogeographic origins and to estimate population admixture. SNP genotyping was performed using the Sequenom MassARRAY genotyping platform with iPLEXchemistry according to manufacturer's recommendations.^12^ We used a panel of 105 unlinked AIMs described by Kosoy^13^ to estimate the proportion of WA, EU, and NA genetic ancestry for each of the study subjects. This panel of AIMs consists of 105 unlinked SNPs (Table S1). In our study population, the mean and median estimated NA genetic ancestry was only 3% and 2%, respectively, and NA ancestry was not included in the analyses.

In the statistical analyses, we first conducted a regression tree analysis to find the cut points of WA percent ancestry in predicting cancer status (cancer on biopsy). A three‐category variable of WA percent ancestry (0.29‐0.79, 0.79‐0.87, and 0.87‐0.99) was obtained as having the most predictive power of cancer status (Figure S1). We then conducted multivariate analyses to predict cancer on biopsy or high‐grade cancer on biopsy using these categorical variables along with three established clinical variables (age, PSA, and prostate volume) as the predictors. First, we fit the logistic regression using the three WA percent ancestry categories alone (with 0.29‐0.79 as the reference group) and then we added the three clinical variables into the model. A joint test of predicting cancer status for both the 0.79‐0.87 and the 0.87‐0.99 categories was also conducted. Finally, the receiver operating characteristic (ROC) analyses were performed using these predictors.

## 3 RESULTS

The demographic and clinical characteristics of the study population are shown in Table 1. Of the 96 consecutive eligible AA study subjects, 49 (51%) were diagnosed with PrCa on a standard‐of‐care ultrasound‐guided 12 core systematic biopsy. The overall Gleason grade was Gleason Sum (GS) 6 for 21 and GS 7‐10 for 28 patients. Age at biopsy was not significantly different between the cancer‐positive and cancer‐negative patient groups or between the cancer‐negative, GS 6 cancer, and GS 7‐10 patient groups. Median prebiopsy PSA levels were not significantly different between the cancer‐positive and cancer‐negative patient groups or between the cancer‐negative, GS 6 cancer, and GS 7‐10 patient groups. One patient with PSA of 2196 ng/mL was removed from analyses involving PSA. The median prostate volume was 35.8 and 58.8 cc in the cancer‐positive and cancer‐negative groups and this difference was significant (*P* = .007) and consistent with benign enlargement of the gland in the PrCa‐negative group. PSA density (PSA/prostate volume) is used to help correct for PSA increases due to benign prostatic enlargement, the median PSA density was 0.16 and 0.12 in the cancer‐positive and cancer‐negative groups, respectively, and this difference was significant (*P* = .011). The overall GS corresponded well with the National Comprehensive Cancer Network (NCCN) criteria for PrCa risk after biopsy, with all but one of the 21 men with GS 6 cancer meeting NCCN criteria for low‐ or very low‐risk PrCa. NCCN criteria for PrCa risk include tumor volume, clinical stage, and prebiopsy PSA as well as GS.

**Table 1:** Demographic and clinical characteristics of self‐identified African American study subjects

	All Subjects	No Cancer on Biopsy	Diagnosed with GS 6 Cancer	Diagnosed with GS 7‐10 Cancer
Number of Subjects	96	47	21	28
Age at biopsy				
Mean (SD)	61.1 (7.6)	61.9 (7.0)	60.6 (9.0)	60.2 (7.6)
Median	61.5	63.0	61.0	60.5
Gleason Sum (GS)				
Grade Group 1	21	na	21	0
Grade Group 2	16	na	0	16
Grade Group 3	5	na	0	5
Grade Group 4‐5	7	na	0	7
NCCN Risk				
Low or very low	20	na	20	0
Intermediate	20	na	1	19
High	9	na	0	9
Serum PSA (ng/mL)^a^				
Mean (SD)	7.84 (7.40)	7.03 (6.10)	5.53 (2.37)	11.03 (10.57)
Median	5.60	5.55	5.58	6.20
Prostate Size (cc)^b^				
Mean (SD)	49.0 (33.7)	58.8 (41.6)	41.7 (19.3)	38.6 (21.7)
Median	40	48.0	37.0	34.6
PSA Density^c^				
Mean (SD)	0.21 (0.23)	0.16 (0.19)	0.15 (0.09)	0.33 (0.31)
Median	0.13	0.12	0.12	0.21

a One man with a PSA of 2196 ng/mL is excluded.

b For one man, prostate volume was missing from the clinical record.

c One man with a PSA of 2196 ng/mL and a second man for whom prostate volume was missing are excluded.

**Figure 1:**
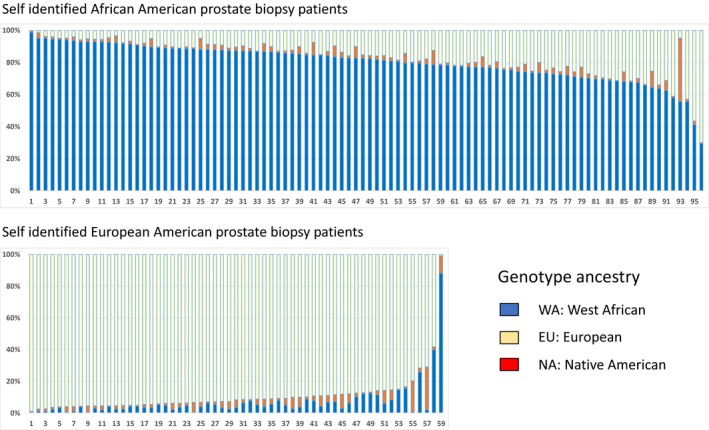
AIMs estimates of individual WA, EU, and NA ancestry for 96 self‐identified African American and 59 self‐identified European American patients undergoing standard‐of‐care systematic prostate biopsy. The mean percentage of WA genetic ancestry for the AA men was 80% (SD 12%) and for the EA men was 7% (SD 12%), and the means were significantly different *P* < .0001. The mean percentage of EA ancestry was 89% (SD 14%) for the EA men and 17% (SD 12%) for the AA men, and the means were significantly different *P* < .0001. The mean percentage of NA ancestry was 3% (SD 4%) for AA men and 4% (SD 4%) for EA men (no significant difference). NA ancestry was not included in the analyses

**Figure 2:**
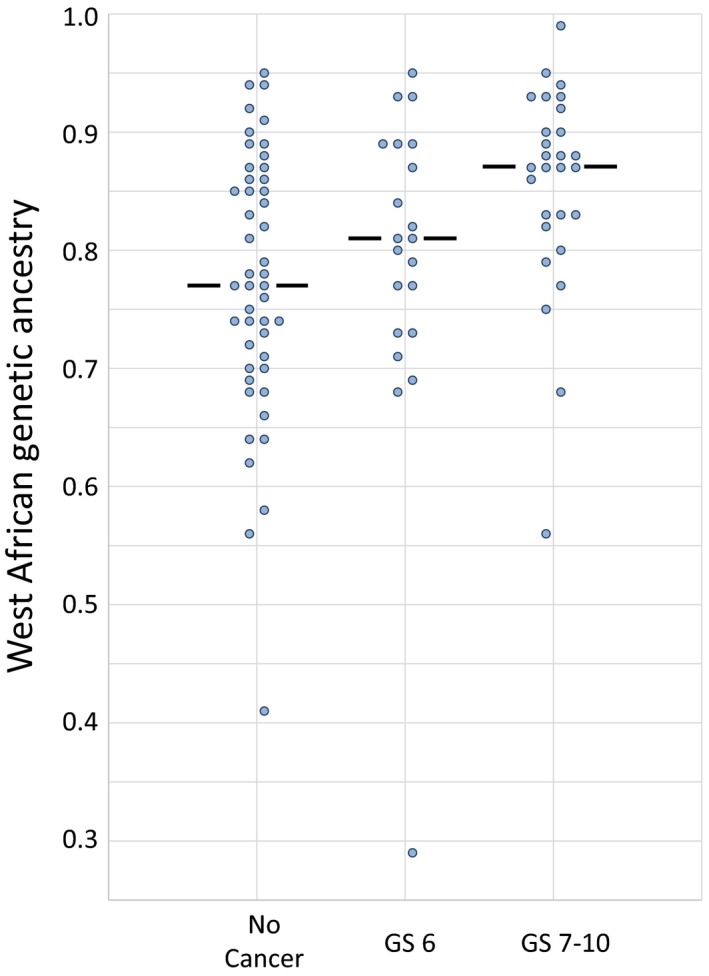
Distribution of WA ancestry in African American men diagnosed with no cancer, low‐grade cancer (GS = 6), and higher grade cancer (GS > 6) on standard biopsy. Median indicated by the horizontal lines

**Figure 3:**
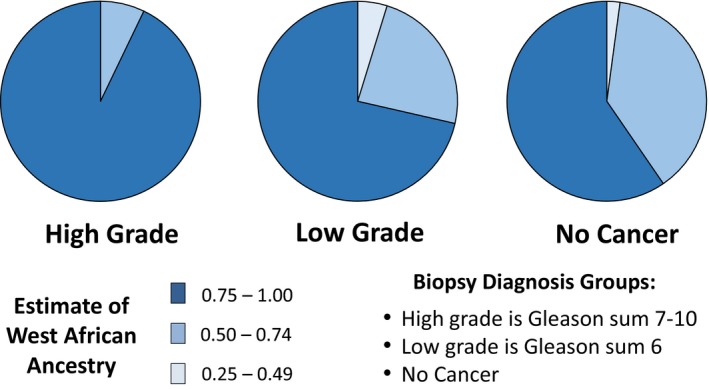
Self‐identified African American prostate biopsy patients (Standard Biopsy): Quartile distribution of AIMs estimates of individual WA ancestry in patients diagnosed with higher grade (GS 7‐10), low‐grade (GS 6), and no PrCa

Ancestry genotyping showed the expected admixture of WA and EU ancestry Figure 1. For the entire group of 99 self‐identified AA patients, the mean estimated WA ancestry was 0.80. There were significant differences in the estimated WA ancestry between the diagnosis groups Figures 2 and 3, Tables 2 and 3. There was a significant difference (*P* < .01) between the estimated WA ancestries of the men who were biopsy‐positive for cancer (0.83) and the men who were cancer‐negative (0.77). Importantly, there was also a significant difference between the men diagnosed with GS 7‐10 PrCa (0.85) and the men diagnosed with GS 6 or no cancer (*P* < .01).

**Table 2:** Summary of WA ancestry in the prostate biopsy diagnosis groups

	All Subjects	Cancer‐negative	Cancer‐positive	GS 6 cancer	GS 7 or more cancer	NCCN very low or low risk	NCCN intermediate or high risk
N	96	47	49	21	28	19	30
Mean WA	0.80	0.77	0.83	0.79	0.85	0.78	0.85
Std Dev	0.12	0.11	0.12	0.14	0.09	0.14	0.09
Median WA	0.82	0.77	0.86	0.81	0.87	0.81	0.87
Std Error	0.01	0.02	0.02	0.03	0.02	0.03	0.02
Min WA	0.29	0.41	0.29	0.29	0.56	0.29	0.56
Max WA	0.99	0.95	0.99	0.95	0.99	0.95	0.99

**Table 3:** Comparison of WA ancestry in AA men diagnosed with no PrCa, low‐grade PrCa (GS = 6), and higher grade PrCa (GS 7‐10) on standard biopsy. Comparison of WA ancestry in AA men diagnosed with PrCa who met criteria for very low/low and intermediate/high NCCN PrCa risk groups

Group 1	Group 2	Two‐tailed *P* value
**Cancer‐Negative**	**Cancer‐Positive**	.0071**
Cancer‐Negative	GS 6 Cancer	.3632
**Cancer‐Negative**	**GS 7 or higher Cancer**	.0011**
GS 6 Cancer	GS 7 or higher Cancer	.0550
**Cancer‐Negative or GS 6 Cancer**	**GS 7 or higher Cancer**	.0015**
Cancer‐Negative	NCCN very low or low risk	.5286
**Cancer‐Negative**	**NCCN intermediate or high risk**	.0008**
Mann‐Whitney Test of Difference of Median WA Ancestry		<.01**

Differences were considered statistically significant if *P* value less than 0.01 (bold type).

After Bonferroni correction for multiple comparisons the comparisons noted by ** remain significant.

**Figure 4:**
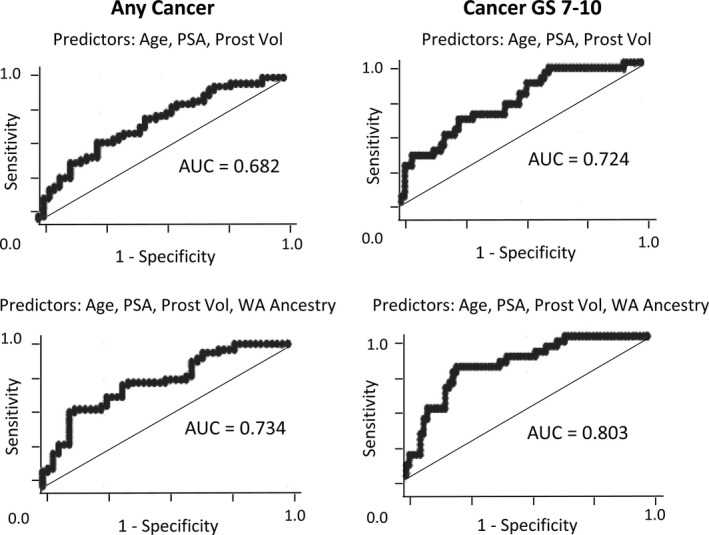
Receiver operating characteristic (ROC) curves comparing the base model (age, PSA, and prostate volume) with a model in which WA ancestry is added to the base model for prediction of any cancer on biopsy and for prediction of GS 7‐10 PrCa on biopsy

% WA ancestry was evaluated as a predictor of GS 7‐10 cancer in our AA subjects both as a single variable and in combination with age, PSA, and prostate size Figure 4 and Table 4. As a single variable, % WA ancestry was a significant predictor of cancer on biopsy, with the AA men of % WA ancestry greater than 87% showing a 4.6 higher odds of PrCa (*P* = .004) and 5.7 higher odds of GS 7‐10 cancer (*P* = .004) than the AA men with greater than 20% admixture. AA men with % WA ancestry between 80% and 87% also showed increased PrCa risk compared to the AA men with greater than 20% admixture, with 2.8 higher odds for any cancer (*P* = .05) and 3.7 higher odds for GS 7‐10 cancer (*P* = .04) than the AA men with greater than 20% admixture. For prediction of any cancer on biopsy, addition of WA to a base model that includes age, PSA, and prostate volume increases the model pseudo R square by 75% with *P* = .0245 for the joint test for two WA categorical variables. For predicting GS 7‐10 cancer, addition of WA ancestry to the base model improves pseudo R square by 72% with *P* = .0238 for the joint test.

**Table 4:** Relationship between AIMs estimate of West African genetic ancestry and the probability of incident prostate on standard biopsy in self‐identified African American (AA) patients. WA ancestry cut points between prostate cancer risk categories were determined by a regression tree analysis and odds ratios for category 3 (WA ancestry 0.872‐0.990) and category 2 (WA ancestry 0.795‐0.871) were determined relative to the reference group, category 1 with greater than 20% ancestry admixture (WA ancestry 0.294‐0.790). For prediction of a diagnosis of cancer on biopsy, addition of WA to a base model that includes age, PSA, and prostate volume increases the model pseudo R square by 75% with *P* = .0245 for the joint test for two WA categorical variables. For predicting GS 7‐10 cancer, addition of WA ancestry to the base model improves pseudo R square by 72% with *P* = .0238 for the joint test

Odds Ratios and *P* Values for Cancer on Biopsy and GS 7‐10 Cancer on Biopsy								
	Mean	Range	Cancer on Biopsy			GS 7‐10 Cancer on Biopsy		
			OR and *P* value^a^	95% CI	AUC (95% CI)	OR and *P* value^a^	95% CI	AUC (95% CI)
Model 1: Logistic regression on WA categorical variables^b^								
WA 0.795‐0.871	0.274	0.0‐1.0	**2.832 (0.0457)**	1.020, 7.865		**3.706 (0.0380)**	1.075, 12.771	
WA 0.872‐0.990	0.305	0.0‐1.0	**4.615 (0.0035)**	1.651, 12.901	0671 (0.563, 0.770)	**5.687 (0.0042)**	1.732, 18.676	0.686 (0.572, 0.793)
Model 2: Multivariate logistic regression: age, PSA, prostate volume, WA ancestry								
Age at biopsy (years)	61.1	38.0‐81.0	1.009 (0.7839)	0.948, 1.074		1.011 (0.739)	0.946, 1.082	
PSA (ng/mL)	7.84	0.67‐46.03	1.049 (0.2223)	0.971, 1.133		**1.115 (0.021)**	1.017, 1.224	
Prostate volume (cc)	49.2	16.0‐239.0	**0.978 (0.0181)**	0.960, 0.996		0.981 (0.079)	0.959, 1.002	
WA 0.795‐0.871	0.274	0.0‐1.0	2.939 (0.0547)	0.979, 8.823		**4.625 (0.032)**	1.144, 18.695	
WA 0.872‐0.990	0.305	0.0‐1.0	**4.306 (0.0100)**	1.418, 13.072	0.7340 (0.633, 0.835)	**6.339 (0.008)**	1.638, 24.531	0.803 (0.708, 0.898)

N = 95, one patient with PSA = 2196 ng/mL was deleted from the analysis.

a ORs with *P* values less than 0.05 were considered statistically significant (bold type).

b The reference group for WA ancestry is the AA subjects whose WA ancestry was less than 0.79.

## 4 DISCUSSION

The factors involved in increased risk for PrCa in AAs have generally been considered in comparison with EAs. In this study, we have focused on differences in potential PrCa risk factors within a population of self‐identified AA men. % WA ancestry varies within the AA population and is higher for AAs in the southern United States than for AAs in the North and West.^14^ Our measures of PrCa risk are focused on the prostate biopsy, and specifically on the pathology diagnosis that is critical to guiding decisions about patient care. We found that WA ancestry was significantly higher in men who were diagnosed with PrCa on biopsy, compared to men who were cancer‐negative, and highest in men who were diagnosed with GS 7‐10 PrCa.

Because of the importance of biopsy GS in assessing cancer severity and thus in determining whether or not a patient should consider AS, we evaluated % WA ancestry as a predictor of GS 7‐10 cancer in our AA subjects both as a single variable and in combination with other variables used clinically to make decisions about whether a patient should undergo prostate biopsy. For prediction of any cancer on biopsy, addition of WA to a base model that includes age, PSA, and prostate volume increases the model pseudo R square by 75% with *P* = .0245. For predicting GS 7‐10 cancer, addition of WA ancestry to the base model improves pseudo R square by 72% with *P* = .0238. Thus when considered in combination with age, PSA, and prostate size, % WA ancestry remained a strong predictor of both PrCa diagnosis and GS 7‐10 cancer on biopsy.

Interestingly, in a study of 244 AA men from Chicago, Kittles and coworkers also found that % WA ancestry was associated with a higher risk of cancer on standard 12 core biopsy, but did not find an association between % WA ancestry and Gleason sum.^15^ A subsequent study of 287 Chicago‐area prostate biopsy patients found that WA ancestry was not predictive of cancer or GS 7‐10 cancer on biopsy.^16^ Both of the Chicago studies used the same panel of ancestry genetic markers as used in our study of patients from Birmingham Alabama and, as expected, the median WA ancestry observed in the Birmingham patients (median 82.2, IQR 73.5‐88.4) is greater than the median reported in the Chicago patients (median 78.4, IQR 69.7‐92.2 and median 80.4; IQR 70.4‐86.4 for Nyame et al^15^ and Nettey et al^16^ respectively). The difference between the association of WA ancestry and higher grade PrCa in the Birmingham and Chicago studies may reflect the limitations of study size. It is also possible that the association between WA ancestry and higher grade incident cancer in Birmingham reflects a greater prevalence of population‐specific genetic or nongenetic factors that contribute to the development of more aggressive disease. Census records indicate that the place of birth for the majority of AAs in the Birmingham AL area is Alabama or Georgia, while AAs from Chicago have come from many different parts of the US and potentially are more genetically diverse. Lachance et al.^17^ report that a relatively small number of genetic loci appear to drive elevated PrCa risk in men of WA descent. Comparing the relative prevalence of PrCa risk loci in AAs in Birmingham and Chicago might identify subtypes of PrCa that are more common in some AA communities than in others. In addition, an examination of potential interactions between WA ancestry and nongenetic risk factors, like obesity that are more prevalent in AAs in the South than in other parts of the US^18^ may reveal modifiable variables that can be incorporated into interventions. Access to care may also have a role in how PrCa genetic risk factors impact AA men. It should be noted that the higher levels of breast cancer‐specific mortality in Latina women with higher NA genetic ancestry that was observed in a population‐based study of patients from San Francisco and Northern California^19^ was not observed in Latina women from the Kaiser Permanente Northern California managed care health system.^20^

One limitation to using prostate biopsies to evaluate PrCa risk is that standard‐of‐care systematic biopsies sample only a small portion of the gland and cancers can be missed. However, the impact of this inherent sampling error is likely to be similar across all of the study subjects. Current studies utilizing MR image‐guided prostate biopsies that reduce sampling error are important for further evaluations of WA ancestry and incident PrCa. If the association between WA ancestry and risk of incident PrCa is confirmed, then incorporating WA ancestry into the guidelines for making decisions about when to biopsy and whether to choose AS may allow AA men to personalize their approach to PrCa screening and management. The commercial success of direct‐to‐consumer ancestry genotyping services suggests that the technical infrastructure is in place to make such a test widely available. What is needed now are the data essential to integrating genetic ancestry with other PrCa risk factors in order to determine how best to use this information to improve patient care.

## CONFLICTS OF INTEREST

None.
