# Supplementary material for: Self‐Identified African Americans and prostate cancer risk: West African genetic ancestry is associated with prostate cancer diagnosis and with higher Gleason sum on biopsy
Source: Cancer Med. 2019 Sep 30;8(16):6915–22. doi: 10.1002/cam4.2434 (PMC6853835; doi:10.1002/cam4.2434)
Supplement: Supplementary file 2 [file CAM4-8-6915-s002.docx]

Supplemental Table 1. Single Nucleotide Polymorphisms (SNPs) used as Ancestry Informative Markers (AIMs)
